# Supplementary material for: Combinatorial chromatin dynamics foster accurate cardiopharyngeal fate choices
Source: eLife. 2019 Nov 20;8:e49921. doi: 10.7554/eLife.49921 (PMC6952182; doi:10.7554/eLife.49921)
Supplement: Transparent reporting form [file elife-49921-transrepform.docx]

***eLife’s* transparent reporting form**

We encourage authors to provide detailed information *within their submission* to facilitate the interpretation and replication of experiments. Authors can upload supporting documentation to indicate the use of appropriate reporting guidelines for health-related research (see [EQUATOR Network](http://www.equator-network.org/%20)), life science research (see the [BioSharing Information Resource](https://biosharing.org/)), or the [ARRIVE guidelines](http://www.plosbiology.org/article/info:doi/10.1371/journal.pbio.1000412) for reporting work involving animal research. Where applicable, authors should refer to any relevant reporting standards documents in this form.

If you have any questions, please consult our Journal Policies and/or contact us: [editorial@elifesciences.org](mailto:editorial@elifesciences.org).

**Sample-size estimation**

- You should state whether an appropriate sample size was computed when the study was being designed
- You should state the statistical method of sample size computation and any required assumptions
- If no explicit power analysis was used, you should describe how you decided what sample (replicate) size (number) to use

Please outline where this information can be found within the submission (e.g., sections or figure legends), or explain why this information doesn’t apply to your submission:

Although no sample size calculation was done prior to experiment, we aimed for sample sizes ranging between 90 and 150 whenever possible for whole mount in situ hybridization and reporter gene assays. With these number of samples scored, we were able to detect statistically and significantly different means between control and treated samples. We indicated the number of embryos scored in the figures and in the corresponding figure legends.

For ATAC-seq, we considered to prepare at least 3 biological replicates to use for further statistical analysis.

**Replicates**

- You should report how often each experiment was performed
- You should include a definition of biological versus technical replication
- The data obtained should be provided and sufficient information should be provided to indicate the number of independent biological and/or technical replicates
- If you encountered any outliers, you should describe how these were handled
- Criteria for exclusion/inclusion of data should be clearly stated
- High-throughput sequence data should be uploaded before submission, with a private link for reviewers provided (these are available from both GEO and ArrayExpress)

Please outline where this information can be found within the submission (e.g., sections or figure legends), or explain why this information doesn’t apply to your submission:

ATAC-seq experiments were performed on 3 or 4 biological replicates. Libraries were removed if they had fewer than 500,000 reads. Bulk RNA-seq experiments were performed on two biological replicates. All sequencing data were deposited on GEO with accession GSE126691.

To review GEO accession GSE126691:
Go to [https://urldefense.proofpoint.](https://urldefense.proofpoint.com/v2/url?u=https-3A__www.ncbi.nlm.nih.gov_geo_query_acc.cgi-3Facc-3DGSE126691&d=DwIBAg&c=slrrB7dE8n7gBJbeO0g-IQ&r=_f_vTVK0I7qGQlcsO2ahOQ&m=dD0hL9y9wAH4WKKa0dyXgN8SxcJ4qXZXEVpeqy6fYAo&s=A6rf38095DrIUyjKZbJ5RuOUs8Kt_nctExxnKBJNIJ8&e=)
Enter token ahepyscebrkrlgx into the box

**Statistical reporting**

- Statistical analysis methods should be described and justified
- Raw data should be presented in figures whenever informative to do so (typically when N per group is less than 10)
- For each experiment, you should identify the statistical tests used, exact values of N, definitions of center, methods of multiple test correction, and dispersion and precision measures (e.g., mean, median, SD, SEM, confidence intervals; and, for the major substantive results, a measure of effect size (e.g., Pearson's r, Cohen's d)
- Report exact p-values wherever possible alongside the summary statistics and 95% confidence intervals. These should be reported for all key questions and not only when the p-value is less than 0.05.

Please outline where this information can be found within the submission (e.g., sections or figure legends), or explain why this information doesn’t apply to your submission:

Statistical tests for differential accessibility are described in Materials & Methods under “Differential accessibility analysis.” Details for GSEA are included in Materials & Methods under “ Gene Set Enrichment Analysis (GSEA).” GSEA is further explained in the Supplementary Text. The statistical tests for motif enrichment are described in Materials & Methods under “Motif analysis of ATAC-seq.” Statistical tests for differential expression are described in Materials & Methods under “Cell-type-specific gene sets.” All other statistical tests are described in the figure legends. Throughout the manuscript we use “FDR” to refer to p-values corrected using the Benjamini-Hochberg method (AKA false discovery rate). All other p-values are unadjusted. Due to the large number of variables tested, we only show the effect size and whether the effect is considered significant. We believe that this gives a more informative picture of the data than the exact p-values, and allows us to more effectively group the data for downstream analysis. For expression and accessibility experiments, we show the effect size as log2 fold change. For motif enrichment, we show effect size as log2 odds ratios (as described in the Supplementary Text). For GSEA, we show effect size as Normalized Enrichment Score (as described in the supplement).

(For large datasets, or papers with a very large number of statistical tests, you may upload a single table file with tests, Ns, etc., with reference to sections in the manuscript.)

**Group allocation**

- Indicate how samples were allocated into experimental groups (in the case of clinical studies, please specify allocation to treatment method); if randomization was used, please also state if restricted randomization was applied
- Indicate if masking was used during group allocation, data collection and/or data analysis

Please outline where this information can be found within the submission (e.g., sections or figure legends), or explain why this information doesn’t apply to your submission:

Sample groups for differential accessibility are described in Materials & Methods under “Differential accessibility analysis.” Peak sets for downstream analysis are defined in Materials & Methods under “Cell-type-specific accessibility.” Sample groups for differential expression and gene sets for downstream analysis are defined in Materials & Methods under “Cell-type-specific expression gene sets.”

**Additional data files (“source data”)**

- We encourage you to upload relevant additional data files, such as numerical data that are represented as a graph in a figure, or as a summary table
- Where provided, these should be in the most useful format, and they can be uploaded as “Source data” files linked to a main figure or table
- Include model definition files including the full list of parameters used
- Include code used for data analysis (e.g., R, MatLab)
- Avoid stating that data files are “available upon request”

Please indicate the figures or tables for which source data files have been provided:

All scripts for data analysis are available at

[https://github.com/ChristiaenLab/ATAC-seq](https://github.com/ChristiaenLab/ATAC-seq)
